# Protein nanoparticles assemble in plants, display antigenic viral peptides, and produce an epitope‐specific immune response

**DOI:** 10.1111/febs.70288

**Published:** 2025-11-17

**Authors:** Jordan T. VanderBurgt, Richard Strasser, Hong Zhu, Angelo Kaldis, Christopher P. Garnham, Rima Menassa

**Affiliations:** ^1^ London Research and Development Centre Agriculture and Agri‐Food Canada London Canada; ^2^ Biology Department University of Western Ontario London Canada; ^3^ Department of Biotechnology and Food Sciences BOKU University Vienna Austria; ^4^ Biochemistry Department University of Western Ontario London Canada

**Keywords:** lumazine synthase, plant molecular farming, protein nanoparticle, PRRS, subunit vaccine

## Abstract

Porcine reproductive and respiratory syndrome (PRRS) is one of the major diseases affecting the global swine industry. Current methods to control this viral disease are insufficient or pose safety concerns; therefore, there is a need for a safer and more effective vaccine against PRRS. In this study, we designed a chimeric nanoparticle vaccine through genetic fusion of an epitope composed of portions from two PRRS viral proteins, M and GP5, with lumazine synthase from *Aquifex aeolicus*. Transient expression in the leaves of *Nicotiana benthamiana* plants resulted in soluble levels around 0.18 mg·g^−1^ of plant fresh weight. This fusion protein assembles into nanoparticle structures surface‐displaying the PRRS epitope and is efficiently glycosylated with oligomannose N‐linked glycans. A mouse immunization trial was conducted using this protein as well as a previously described protein consisting of the same epitope displayed on a modified tobacco mosaic virus coat protein, and both vaccine candidates induced epitope‐specific antibodies. This study demonstrates the feasibility of protein nanoparticle‐based vaccines against PRRS produced in plants and lays the foundation for future studies to evaluate vaccine efficacy in pigs.

## Graphical abstract

Current methods to control porcine reproductive and respiratory syndrome, a disease affecting pigs, are insufficient and a safer, more effective vaccine is needed. This study describes the design and plant production of a self‐assembling protein nanoparticle vaccine candidate against this disease. The vaccine candidate accumulates well in the plant leaves and assembles into nanoparticles displaying the viral epitope. Two vaccine candidates induced epitope‐specific antibodies when administered to mice.

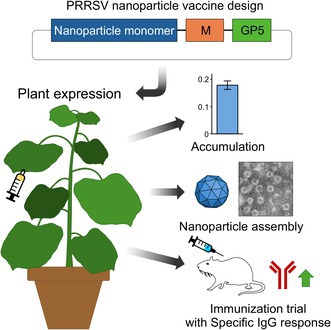

**Abbreviations**
AaLS: lumazine synthase from *Aquifex aeolicus*
AaLSm: modified lumazine synthase from *Aquifex aeolicus*
AMG: vaccine candidate composed of AaLSm and the viral epitope
ELISA: enzyme‐linked immunosorbent assay
ER: endoplasmic reticulum
GP5: glycosylated protein 5 from PRRSV
IMAC: immobilized metal affinity chromatography
LC–MS: liquid chromatography–mass spectroscopy
M: matrix protein from PRRSV
PBS: phosphate‐buffered saline
PRRS: porcine reproductive and respiratory syndrome
PRRSV: porcine reproductive and respiratory syndrome virus
SDS/PAGE: sodium dodecyl‐sulfate polyacrylamide gel electrophoresis
SEC: size‐exclusion chromatography
TEM: transmission electron microscopy
TMG: vaccine candidate composed of TMVc and the viral epitope
TMVc: modified tobacco mosaic virus coat protein
VLP: virus‐like particle

## Introduction

Porcine reproductive and respiratory syndrome (PRRS) is one of the major diseases affecting the global swine industry [1, 2]. This disease, caused by the PRRS virus (PRRSV), leads to sneezing, coughing, pneumonia, stunting of growth, and reduced life expectancy in pigs. Additionally, pregnant sows infected with PRRSV experience high rates of pregnancy complications, including spontaneous abortions and stillbirths [2, 3]. Despite being a viral disease, PRRS contributes to the rising issue of antimicrobial resistance due to the secondary bacterial superinfections that often accompany it, such as *Mycoplasma hyopneumoniae* [4]. To decrease antibiotic use associated with treating these opportunistic infections [5], vaccination against PRRS could be an effective tool in the hands of farmers [6]. More generally, increased vaccination has been described as one of the best alternatives to antimicrobial use in pigs [7].

PRRSV is a small, enveloped RNA virus from the order *Nidovirales* and family *Arteriviridae*. This virus is classified into two distinct species, PRRSV‐1 and PRRSV‐2, which share on average 65% genome sequence identity [8]. PRRSV‐1 is mainly localized to Europe but PRRSV‐2 is prevalent globally and is the focus of this study [9, 10]. While there are several vaccines available against PRRS including live attenuated and inactivated virus vaccines, they are either ineffective at providing cross‐strain protection or pose safety concerns [2, 8, 11]. Therefore, there is currently a need for a safer and more effective vaccine against this disease.

The two most abundant proteins in the PRRSV envelope are the glycosylated protein 5 (GP5) and matrix protein (M) which interact via a disulfide bond between their N‐terminal ectodomains [12]. GP5 and M play an essential role in virion formation and exocytosis from host cells and are also involved in cell attachment and entry [12]. Several PRRSV proteins contain neutralizing antibody binding sites; however, those within GP5 were shown to be the most effective at controlling infection [13, 14, 15]. While the M protein does not contain any neutralizing epitopes, co‐expression of M and GP5 together amplifies the anti‐GP5 immune response [16].

Protein nanoparticles are composed of individual monomers that self‐assemble into stable quaternary structures [17]. Some are based on viral coat proteins and are referred to as virus‐like particles (VLPs) or viral nanoparticles, whereas others originate from bacteria and form smaller cage‐like structures [17, 18]. Regardless of origin, protein nanoparticles are an innovative tool in vaccine design, acting as scaffolds for the display of antigenic peptides [19]. Nanoparticle‐based vaccines show improved interaction with the immune system due to the larger particle size facilitating movement towards lymphoid tissues and repetitive display of epitopes enhancing interaction with immune cells [17].

One protein nanoparticle of interest is the lumazine synthase from *Aquifex aeolicus* (AaLS), a hyperthermophilic bacterium that grows near underwater hot springs [20]. AaLS is an enzyme that catalyzes the penultimate step of riboflavin synthesis [21]. This protein self‐assembles into a 60‐subunit hollow sphere with an external diameter of 15.4 nm [20, 22] and can tolerate the addition of foreign peptides for applications such as vaccine development [19, 23]. Compared to some other protein nanoparticles, such as the lumazine synthase from *Brucella* sp. which forms as a smaller dimer of pentamers [24], the larger size of AaLS assemblies should facilitate efficient trafficking to lymphatic tissues and more copies of an epitope to be displayed [17]. Additionally, AaLS is highly thermostable, with a melting temperature of approximately 120 °C [20] that may aid in downstream processing of AaLS‐based biopharmaceuticals.

Plants are an innovative platform for recombinant protein production, often for pharmaceutical or biotechnological purposes [25, 26]. Plant production has many benefits over other platforms, including versatility, speed, scalability, and low costs [27, 28]. Plants are also unaffected by mammalian pathogens, which is a concern with other platforms such as mammalian cells [27, 29]. As a eukaryotic expression system, plants can facilitate post‐translational modifications such as glycosylation and disulfide bonds which are important for the folding, stability, function, or immunogenicity of some proteins [30, 31].

We have previously demonstrated the plant production and characterization of a VLP‐based PRRS vaccine candidate through genetic fusion of an M‐GP5 epitope to a modified tobacco mosaic virus coat protein (TMVc) [32]. In this study, we describe the plant production and characterization of a PRRS vaccine candidate composed of modified AaLS nanoparticles harboring the M‐GP5 epitope. The vaccine candidate accumulates to relatively high soluble levels within the plant leaves and assembles into nanoparticles displaying the viral epitope. We conducted a mouse immunization trial with these two nanoparticle‐based vaccine candidates and found that both induce epitope‐specific antibody titers in mice.

## Results

### AaLS accumulates in plant leaves

AaLS nanoparticles show promise as a scaffold for vaccine development; however, to our knowledge, this protein had not been produced in plants before. Because our goal is to develop the AaLS nanoparticle for the display of a glycosylated peptide, we expressed it transiently in *Nicotiana benthamiana* leaves and targeted it with a signal peptide to the apoplast, vacuole, and endoplasmic reticulum (ER) where N‐linked glycosylation of proteins occurs. As a bacterial protein, AaLS does not undergo glycosylation in its native bacterial host, but it contains a potential glycosylation site at N102 as determined by NetNGlyc – 1.0 (https://services.healthtech.dtu.dk/services/NetNGlyc‐1.0). Therefore, we needed to evaluate whether AaLS could be expressed and whether it would be glycosylated in the plant secretory pathway.

**Fig. 1:**
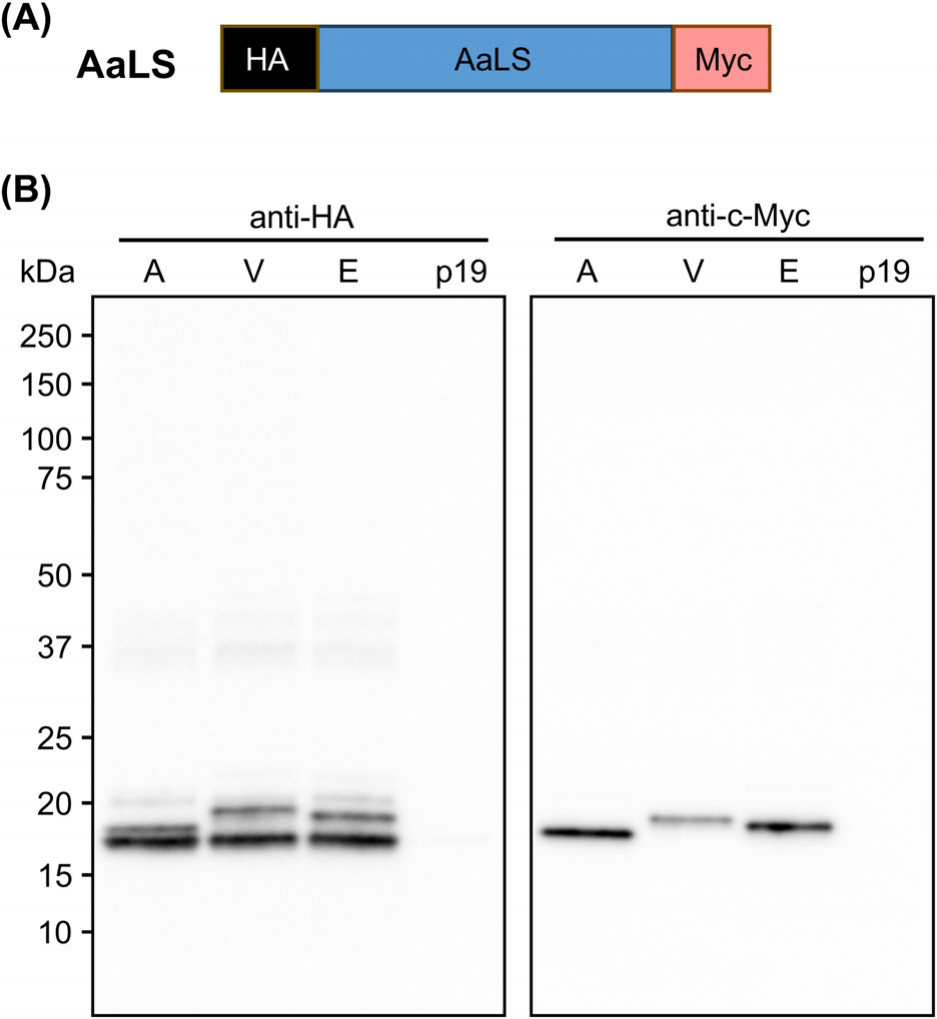
AaLS accumulates when targeted along the secretory pathway. (A) A schematic of the lumazine synthase from *Aquifex aeolicus* (AaLS; blue), flanked by hemagglutinin (HA; black), and c‐Myc tags (Myc; pink). (B) AaLS targeted to the apoplast, vacuole, and ER (A, V, E respectively) with p19 as a negative control. The left and right blots were probed with anti‐HA and anti‐c‐Myc primary antibodies, respectively. The expected molecular weight of AaLS is 18.9 kDa. In each lane, 16 μL of crude extract was loaded corresponding to 1.6 mg of leaf fresh weight. This experiment was repeated twice with similar banding patterns.

Plant extracts expressing the AaLS construct (Fig. 1A) were analyzed by sodium dodecyl‐sulfate polyacrylamide gel electrophoresis (SDS/PAGE) and immunoblotting. Probing with an anti‐HA antibody revealed a doublet of bands when the protein was targeted to the vacuole and the ER (Fig. 1B), and a tight doublet was also present in the sample targeted to the apoplast. In contrast, probing with an anti‐c‐Myc antibody shows one band per sample corresponding in size to the upper band of each anti‐HA‐probed sample. Previous work using these plasmids reported that apoplast‐targeted proteins run smaller than ER‐ or vacuole‐targeted proteins [32]. This is likely due to the C‐terminal KDEL and vacuolar targeting sequences remaining on the mature proteins targeted to the ER and the vacuole, respectively (Fig. 1B). However, the lower band observed upon anti‐HA immunoblotting represents removal of the c‐Myc tag and ER or vacuolar targeting peptides by C‐terminal cleavage of the recombinant protein that may result in mistargeting. Therefore, we decided to proceed with the apoplast‐targeted construct.

### AaLSm and AaLSm‐M‐GP5 accumulate in plants

**Fig. 2:**
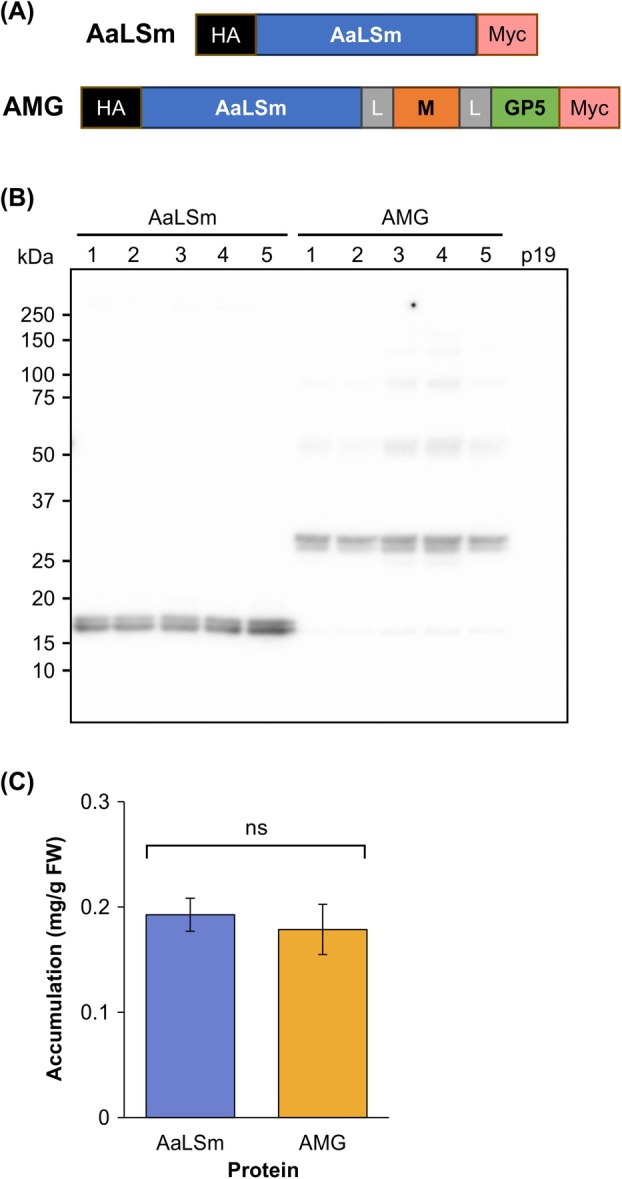
AaLSm‐based recombinant proteins accumulate to relatively high levels in plants. (A) Constructs for the modified lumazine synthase from *Aquifex aeolicus* (AaLSm) and genetic fusion of AaLSm to the developed M‐GP5 epitope from porcine reproductive and respiratory syndrome virus (AMG). AaLSm (blue), M and GP5 ectodomains (orange and green, respectively) are separated by flexible (GGS)_4_ linkers (L; gray). Both constructs are flanked by hemagglutinin (HA; black) and c‐Myc tags (Myc; pink). (B) Western blot of AaLSm and AMG accumulation at 7‐day postinfiltration (dpi). Numbers across the top (1–5) identify different plants as biological replicates, and p19 represents plants infiltrated with p19 only as a negative control. The expected molecular weights of AaLSm and AMG are 18.9 and 24.9 kDa, respectively. In each lane, 10 μL of crude extract were loaded corresponding to approximately 1 mg of plant tissue fresh weight. All proteins were targeted to the apoplast and sampled on 7 dpi. This blot was probed with an anti‐HA primary antibody and is representative of three transient expression experiments. (C) Mean recombinant protein accumulation levels of 15 biological replicates over three independent experiments (five biological replicates per experiment). Accumulation values are shown in milligrams of recombinant protein per gram of plant fresh weight (FW). Error bars represent standard error of the mean. The accumulation between proteins was not significantly different (ns; *P*‐value > 0.05), as determined by a Welch two sample *t*‐test.

Despite a lack of evidence of AaLS being glycosylated when targeted to the plant secretory pathway, we wanted to avoid this possibility altogether as it may affect protein folding and quaternary assembly. Therefore, we introduced a single amino acid substitution at the one potential glycosylation site in the AaLS coding sequence (N102Q), and we called this protein AaLSm (Fig. 2A).

Proteins that reside within cellular membranes, such as the PRRSV M and GP5 proteins, are difficult to produce in their entirety in heterologous systems [33]. Therefore, we focused on the ectodomains which are exposed on the surface of the virus particle. The GP5 ectodomain from PRRSV strain VR‐2332 contains 4 N‐linked glycosylation sites (N30, N34, N44, and N51). N‐linked glycosylation at site N44 of the GP5 ectodomain is required to elicit high neutralizing antibody titers against the native PRRSV [34]. Additionally, glycosylation at N34 and N51 shields the epitope from the immune system, and avoiding glycosylation at these sites improves the resulting immunogenicity [35]. We have previously reported the production of a mutant M‐GP5 fusion epitope (N34A and N51A) [32] and used the same epitope in this study fused to the surface exposed C terminus of AaLSm [23]. Flexible linkers were placed between the elements of this construct, and we refer to this protein as AMG (Fig. 2A).

Upon transient expression and analysis by SDS/PAGE and immunoblotting, AaLSm appeared as a close doublet of bands slightly smaller than the expected size of 18.9 kDa (Fig. 2B). This doublet of bands appears when probed with an anti‐HA antibody; however, only one band is present upon probing with an anti‐c‐Myc antibody (data not shown), as observed with wild‐type AaLS. Therefore, the upper band is the whole AaLSm monomer and the lower band represents cleavage at the C‐terminal c‐Myc tag. AMG shows a similar banding pattern slightly larger than the expected size of 24.9 kDa. As this protein has two potential N‐glycosylation sites within the M‐GP5 epitope (N30 and N44), AMG running larger than anticipated may be the result of glycosylation. The larger faint bands in the fusion protein samples are likely dimers and further assembly products because the sizes appear in multiples of the monomeric weight.

Quantification of 15 biological replicates for each protein (Fig. 2C) shows that AaLSm (0.193 mg·g^−1^, SE 0.0157) and AMG (0.179 mg·g^−1^, SE 0.0238) accumulate to similar levels (*P*‐value = 0.6278). Therefore, genetic fusion of the PRRSV M‐GP5 epitope to AaLSm shows no negative impact on recombinant protein accumulation.

### AaLSm assembles into nanoparticles that can effectively display the PRRSV epitope

**Fig. 3:**
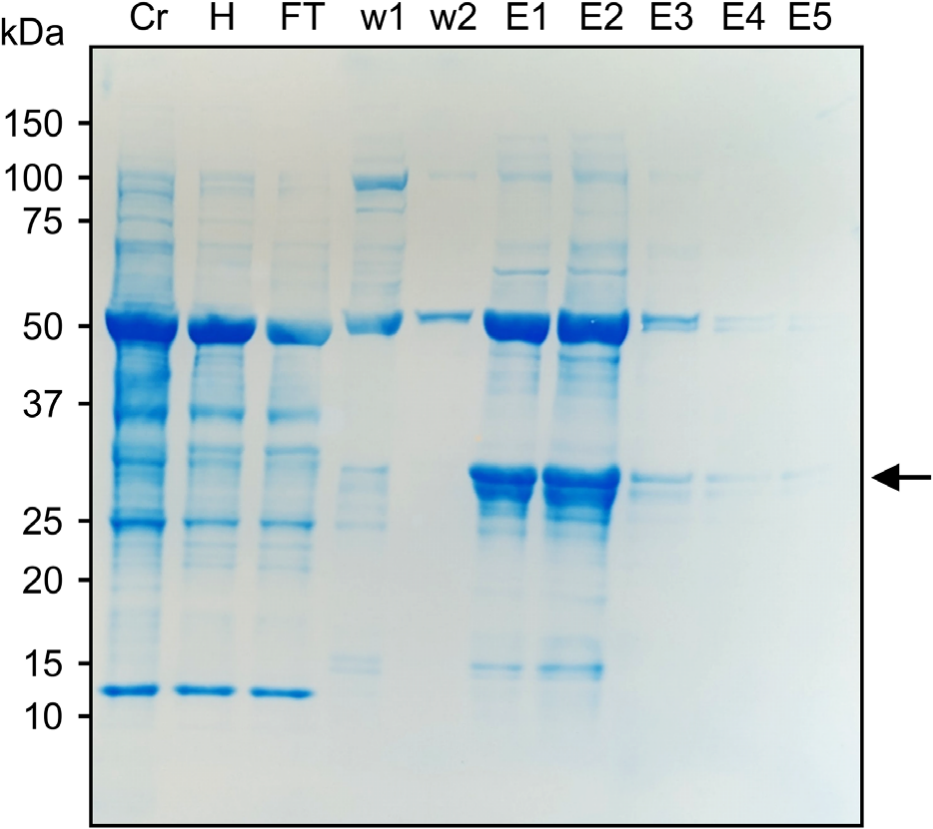
AMG can be effectively purified by IMAC. Stained membrane for immobilized metal affinity chromatography (IMAC) purification of the modified lumazine synthase from *Aquifex aeolicus* with the M‐GP5 epitope from porcine reproductive and respiratory syndrome virus (AMG). Samples include the crude plant extract (Cr) and extract following heat treatment (H) as well as the IMAC flowthrough (FT), washes (w1‐2), and elutions (E1‐5). The arrow represents the expected size of AMG as seen in Fig. 2B. Bands corresponding to AMG are not visible in the Cr or H samples but are one of the most prevalent proteins in elutions 1–2. In each lane, 15 μL of sample was loaded. This figure is a typical representation of IMAC purification of this protein, which was repeated over a dozen times.

Although plants have not previously been used to produce AaLS, different expression systems such as *Escherichia coli* and mammalian cells have been used to produce and assemble AaLS‐based nanoparticles [19, 23]. To determine if *N. benthamiana* can assemble AaLSm nanoparticles, a C‐terminal 6xHis tag was added to both AaLSm and AMG to facilitate purification by immobilized metal affinity chromatography (IMAC). Following transient expression and protein extraction, heat treatment was used to remove some of the endogenous plant proteins as AaLS has high thermostability and should resist denaturation at the applied temperature [20]. Both AaLSm (data not shown) and AMG were then purified by IMAC (Fig. 3). The IMAC‐purified samples were subsequently separated by size‐exclusion chromatography (SEC) to isolate assembled particles.

**Fig. 4:**
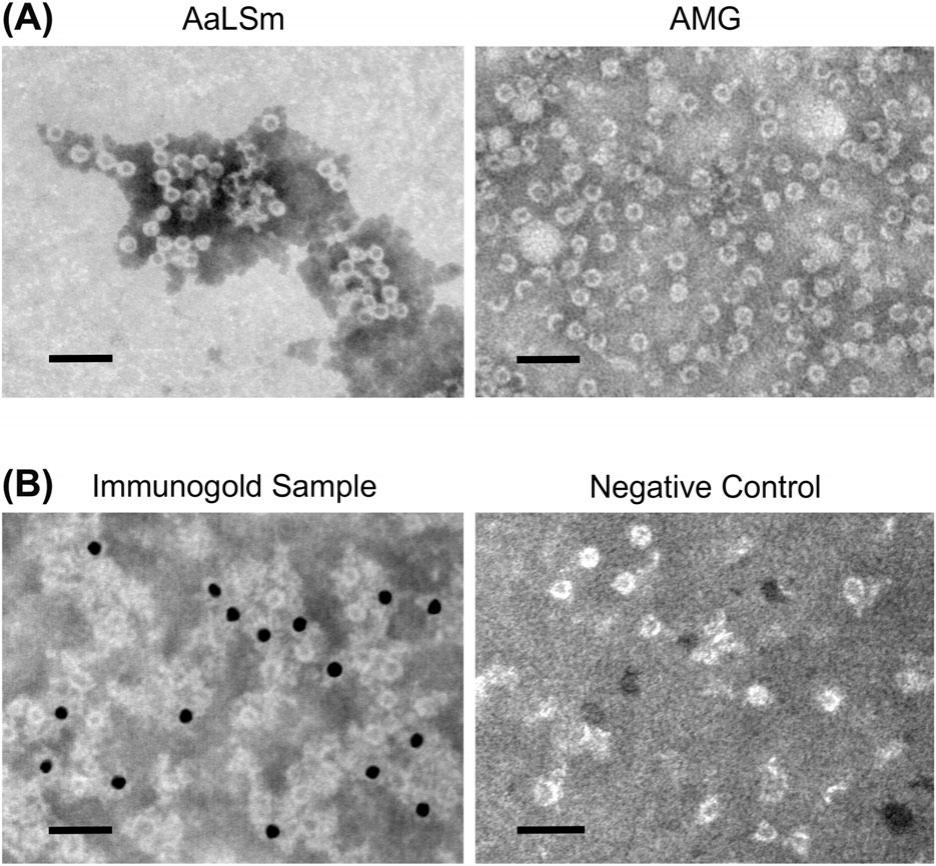
Assembly and epitope display of AaLSm‐based protein nanoparticles. (A) Transmission electron microscopy (TEM) micrographs of the modified lumazine synthase from *Aquifex aeolicus* (AaLSm) and the genetic fusion of AaLSm to the M‐GP5 epitope from porcine reproductive and respiratory syndrome virus (AMG) show similar assembly into nanoparticles around the expected diameter of 15.4 nm. (B) Immunogold localization with AMG using anti‐c‐Myc primary antibodies and 10 nm gold‐conjugated secondary antibodies. Gold particles bound close to the nanoparticles indicate that the M‐GP5 epitope is being surface displayed. The negative control did not include the primary antibody, and nanoparticles are present but no gold particles are visible. Scale bars represent 50 nm. Size‐exclusion chromatography (SEC) purified protein samples were negatively stained and examined using a TEM (JEM‐1400, JEOL) operated at 80 kV. The displayed results are representative of at least 3 separate experiments with consistent observed particle sizes and, for the immunogold localization, the presence or absence of gold particles.

Transmission electron microscopy of purified AaLSm shows spherical assemblies with a diameter around the expected size of 15.4 nm (Fig. 4A), demonstrating successful assembly upon plant expression. Similar purification and visualization of AMG show nanoparticles indistinguishable from those of AaLSm alone (Fig. 4A). Therefore, fusion of the M‐GP5 epitope to the modified AaLSm scaffold does not disrupt quaternary assembly.

Immunogold localization was performed for AMG to assess whether the epitope is effectively displayed on these nanoparticles where it may interact with the immune system. The presence of gold particles in close proximity to the nanoparticles in the experimental sample compared to the control (Fig. 4B) confirms that the M‐GP5 epitope is displayed on the surface of these assemblies.

### Plant‐made AMG and TMG carry mostly oligomannose glycans

**Fig. 5:**
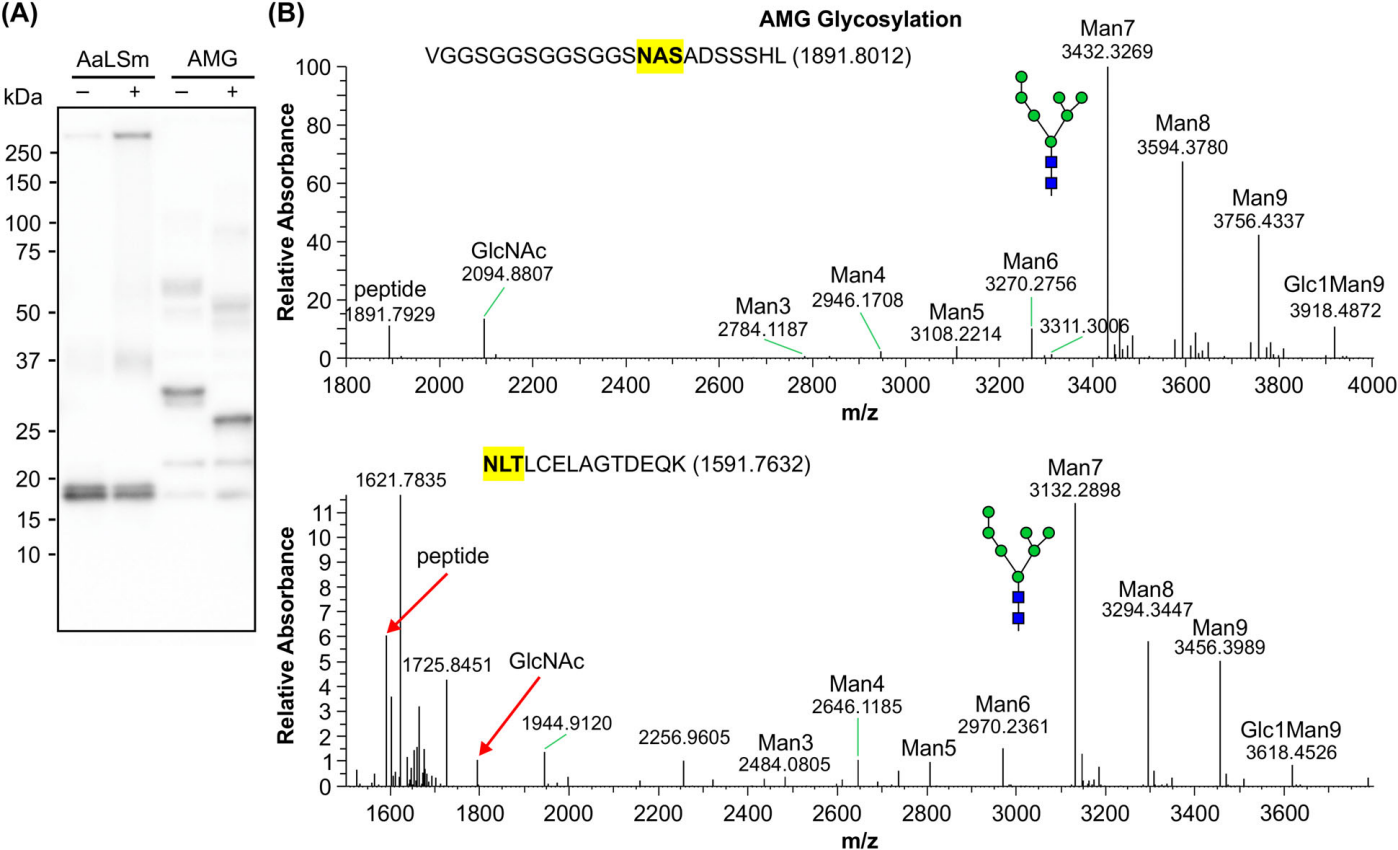
Secreted AMG is efficiently glycosylated with oligomannose *N*‐glycans. (A) Deglycosylation using Peptide:*N*‐glycosidase F (PNGase F) of the modified lumazine synthase from *Aquifex aeolicus* (AaLSm) and the genetic fusion of AaLSm to the M‐GP5 epitope from porcine reproductive and respiratory syndrome virus (AMG), showing both treated (+) and untreated (−) samples. The blot was probed with an anti‐hemagglutinin (HA) primary antibody. (B) Characterization of the glycan composition at two sites along the M‐GP5 epitope sequence of AMG using mass spectrometry. Protein samples were isolated from whole cell lysate. Both *N*‐glycosylation sites (highlighted in yellow, the sequences of the analyzed peptides are shown above the spectra, in NLTLCELAGTDEQK the peptide mass is increased due to carbamidomethylation of the cysteine during the sample preparation) are within the GP5 portion of the epitope and represent sites N30 and N44 along the GP5 ectodomain. The glycan nomenclature and illustration are according to Altmann *et al*. [36], with green circles and blue squares representing mannose (Man) and *N*‐acetylglucosamine (GlcNAc), respectively. This experiment was conducted on two replicates per sample.

To be an effective vaccine against PRRS, the GP5 ectodomain sequence used in this study should be glycosylated [34]. Because we used the same mutant GP5 ectodomain as previously described for altered glycosylation [32], we expected glycosylation at two potential glycosylation sites on the GP5 ectodomain, N30 and N44. Enzymatic deglycosylation using PNGase F of AaLSm and AMG revealed that AaLSm is not glycosylated, while a clear shift in mobility was observed for AMG (Fig. 5A). We had previously reported that deglycosylation of TMVc‐M‐GP5 (TMG) with PNGase F also resulted in a band shift and concluded that the protein is glycosylated. However, because there are two *N*‐glycosylation sites along TMVc, we could not be certain that the GP5 portion of the protein was also glycosylated [32]. Further, as both AMG and TMG should be secreted, it was unexpected for PNGase F to cleave predicted complex glycans carrying α1,3 fucose [37].

**Fig. 6:**
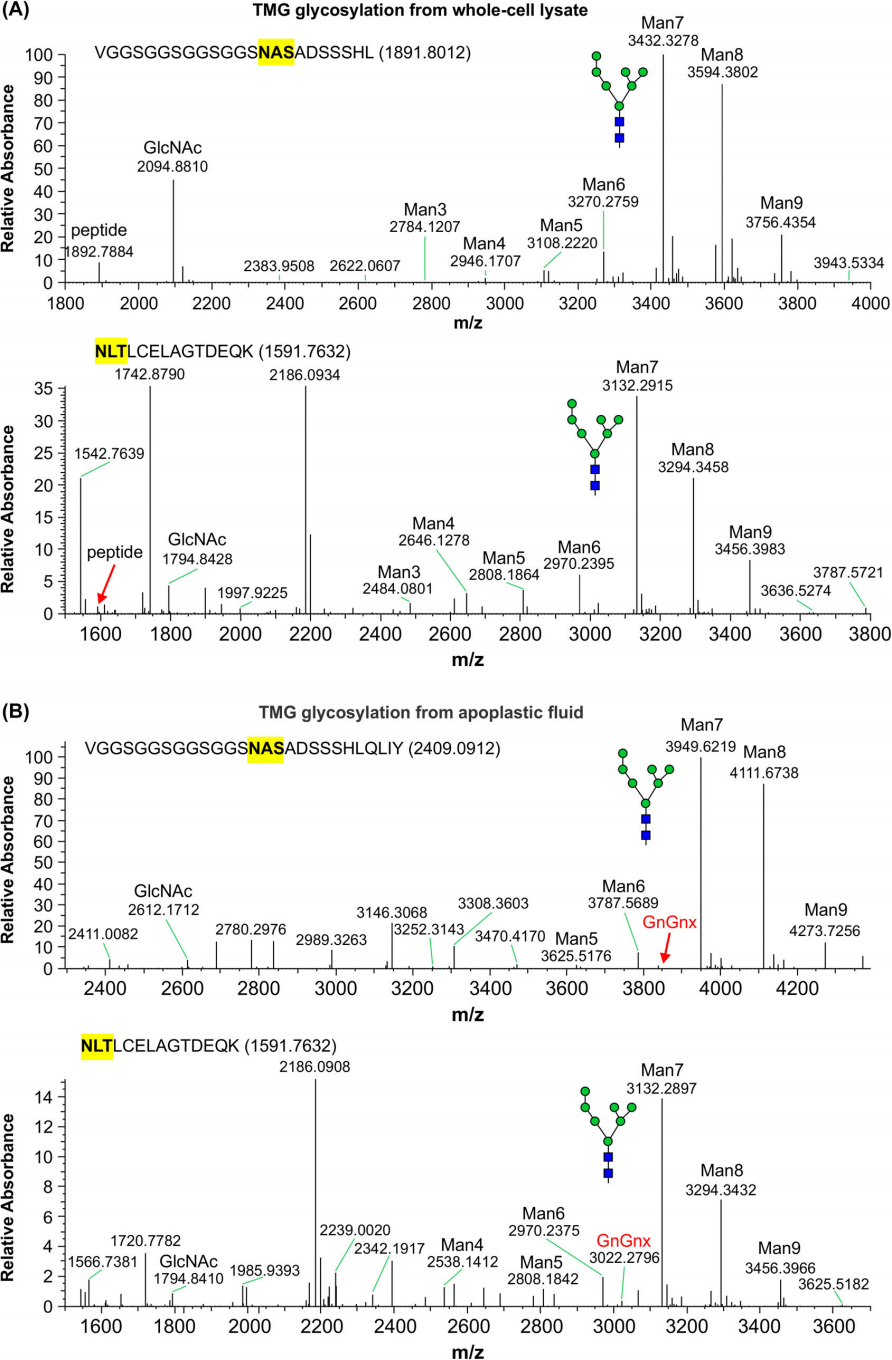
Secreted TMG is efficiently glycosylated with oligomannose *N*‐glycans. Characterization of the glycan composition at two sites along the epitope sequence of the modified tobacco mosaic virus coat protein genetically fused to the M‐GP5 epitope from porcine reproductive and respiratory syndrome virus (TMG). Samples shown are from (A) whole cell lysate and (B) apoplast isolation, using mass spectrometry. Both *N*‐glycosylation sites (highlighted in yellow) are within the GP5 portion of the epitope and represent sites N30 and N44 along the GP5 ectodomain. The sequences of the analyzed peptides are shown above the spectra. In NLTLCELAGTDEQK, the peptide mass is increased due to carbamidomethylation of the cysteine during sample preparation. The small peaks corresponding to the complex *N*‐glycan GnGnX are labeled (red). The glycan nomenclature and illustration are according to Altmann *et al*. [36], with green circles and blue squares representing mannose (Man) and *N*‐acetylglucosamine (GlcNAc), respectively. This experiment was conducted on two replicates for the whole cell lysate and once for the apoplastic fluid.

To further characterize the composition of N‐linked glycans on AMG and TMG, mass spectrometry was performed. Protein samples from whole cell lysates were purified using IMAC and separated by SDS/PAGE. The protein bands were cut from the gel, subjected to proteolytic digestion, and the glycopeptides were subjected to liquid chromatography–mass spectrometry (LC–MS) to obtain the site‐specific *N*‐glycan profile. For both AMG (Fig. 5B) and TMG (Fig. 6A), the two expected sites within the GP5 sequence of the PRRSV epitope were found to be efficiently glycosylated, with only small portions undecorated. Both sites show a similar pattern of glycan composition with mostly oligomannose *N*‐glycans, predominantly Man7 (Man7GlcNAc2). Oligomannose glycans are characteristic of ER‐resident proteins, suggesting that AMG and TMG are either retained in the ER or proceed to the apoplast without glycan processing in the Golgi. To evaluate this further, LC–MS was performed on TMG from an apoplastic fluid isolation (Fig. 6B). The TMG glycan profile from apoplastic fluid is very similar to the whole cell lysate, containing predominantly oligomannose *N*‐glycans. There is, however, slight evidence of Golgi processing as a very small portion of protein has complex *N*‐glycans with β1,2‐xylose present (GnGnX). Therefore, the recombinant proteins appear to be secreted to the apoplast but the N‐linked glycans are not efficiently processed in the Golgi.

### The vaccine candidates induce epitope‐specific antibody responses in mice

**Fig. 7:**
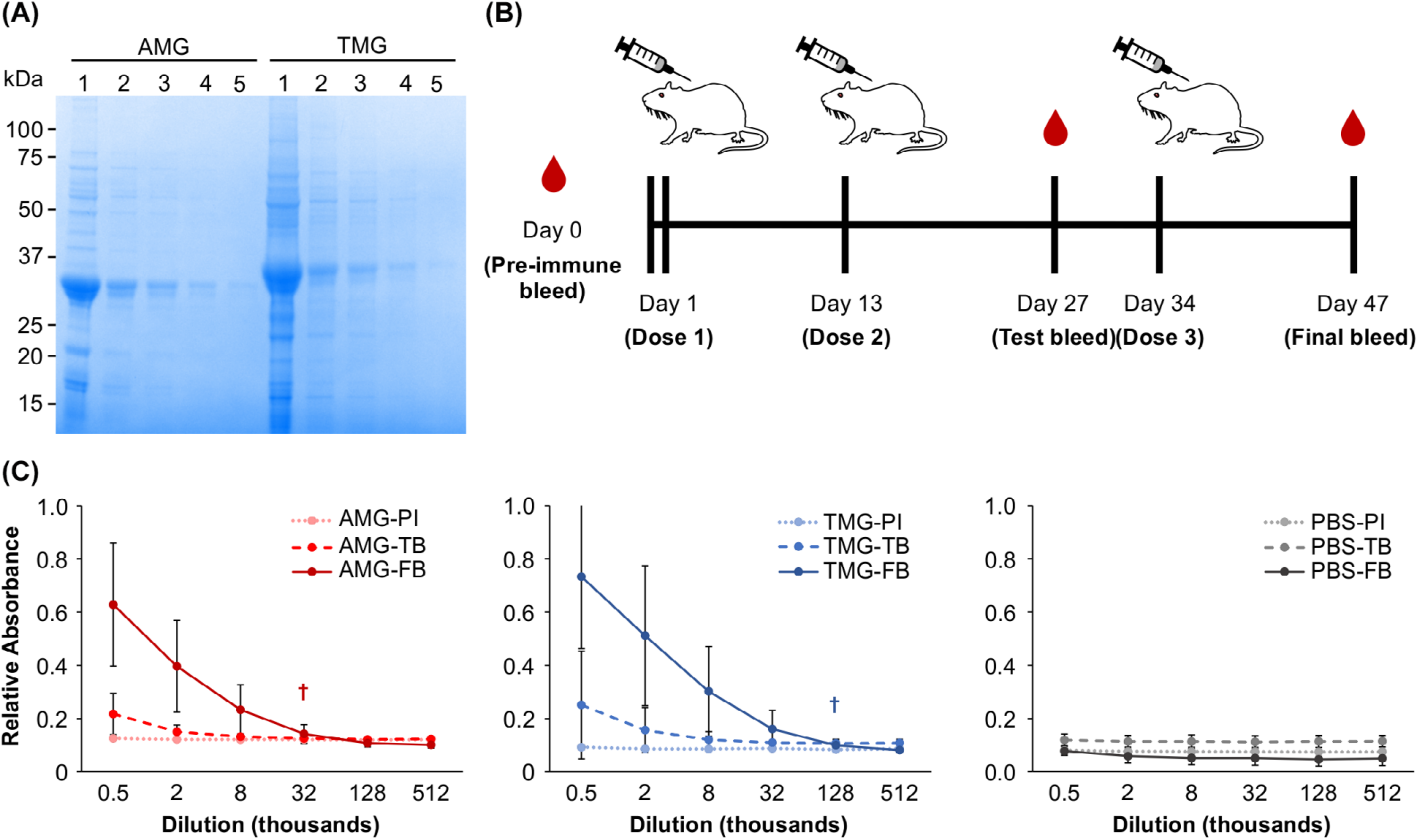
PRRSV vaccine candidates induced epitope‐specific antibody responses in mice. (A) Stained gel for a dilution series of purified vaccine candidate samples for the mouse immunization trial. The samples are composed of the M‐GP5 epitope from porcine reproductive and respiratory syndrome virus (PRRSV) genetically fused to either the modified lumazine synthase from *Aquifex aeolicus* (AMG) or the modified tobacco mosaic virus coat protein (TMG). In each lane, 5 μL of serially diluted protein samples were loaded. Dilutions are as follows: (1) undiluted, (2) 5× dilution, (3) 10× dilution, (4) 20× dilution, and (5) 40× dilution. (B) Timeline for mouse immunization trial. (C) Average enzyme‐linked immunosorbent assay (ELISA) absorbances across a dilution series of mouse sera for the AMG treatment, TMG treatment, and PBS control. For AMG and TMG treatments, the ELISA plates were coated with the alternate protein to evaluate epitope‐specific antibodies. For the PBS control treatment, the data shown is a combination from separate plates coated with AMG and TMG antigen. Absorbance values are relative to a dilution series of a commercial anti‐c‐Myc primary antibody standard to allow for consistency between ELISA plates. Pre‐immune bleeds (PI) are represented by dotted lines, test bleeds (TB) by dashed lines, and final bleeds (FB) by solid lines. † Denote the endpoint titer for the final bleed of each vaccine treatment group. Error bars represent standard error of the mean. Sample sizes of the AMG, TMG, and PBS treatment groups are 12, 11, and 11, respectively.

A mouse immunization trial was performed to assess the vaccine immunogenicity of both AMG and TMG. For each dose, mice were subcutaneously injected with either 33 μg of purified AMG antigen, purified TMG antigen (Fig. 7A), or phosphate‐buffered saline (PBS) as a negative control. The mice received three doses on days 1, 13, and 34 (Fig. 7B). A pre‐immune blood sample was taken from each mouse before the first dose, a test bleed on day 27, and a final bleed on day 47. The mice were monitored for adverse reactions and showed no negative symptoms throughout the trial.

IgG immune responses to the vaccine candidates were determined by enzyme‐linked immunosorbent assay (ELISA) using serial dilutions of the mouse sera. ELISA plates were coated with the cognate antigen to determine the antibody responses to the entire nanoparticle–epitope fusion, while the responses to the M‐GP5 epitope were determined by coating the ELISA plates with the alternate nanoparticle carrying the same epitope.

In the final bleeds, both treatments induced M‐GP5 epitope‐specific antibody responses with endpoint titers of 32 000‐ and 128 000‐times dilution for AMG and TMG, respectively (Fig. 7C). In contrast, the test bleeds showed limited responses indicating that three doses were required to effectively stimulate antibody production. There was no observable response in the control mice injected with PBS throughout the experiment.

**Fig. 8:**
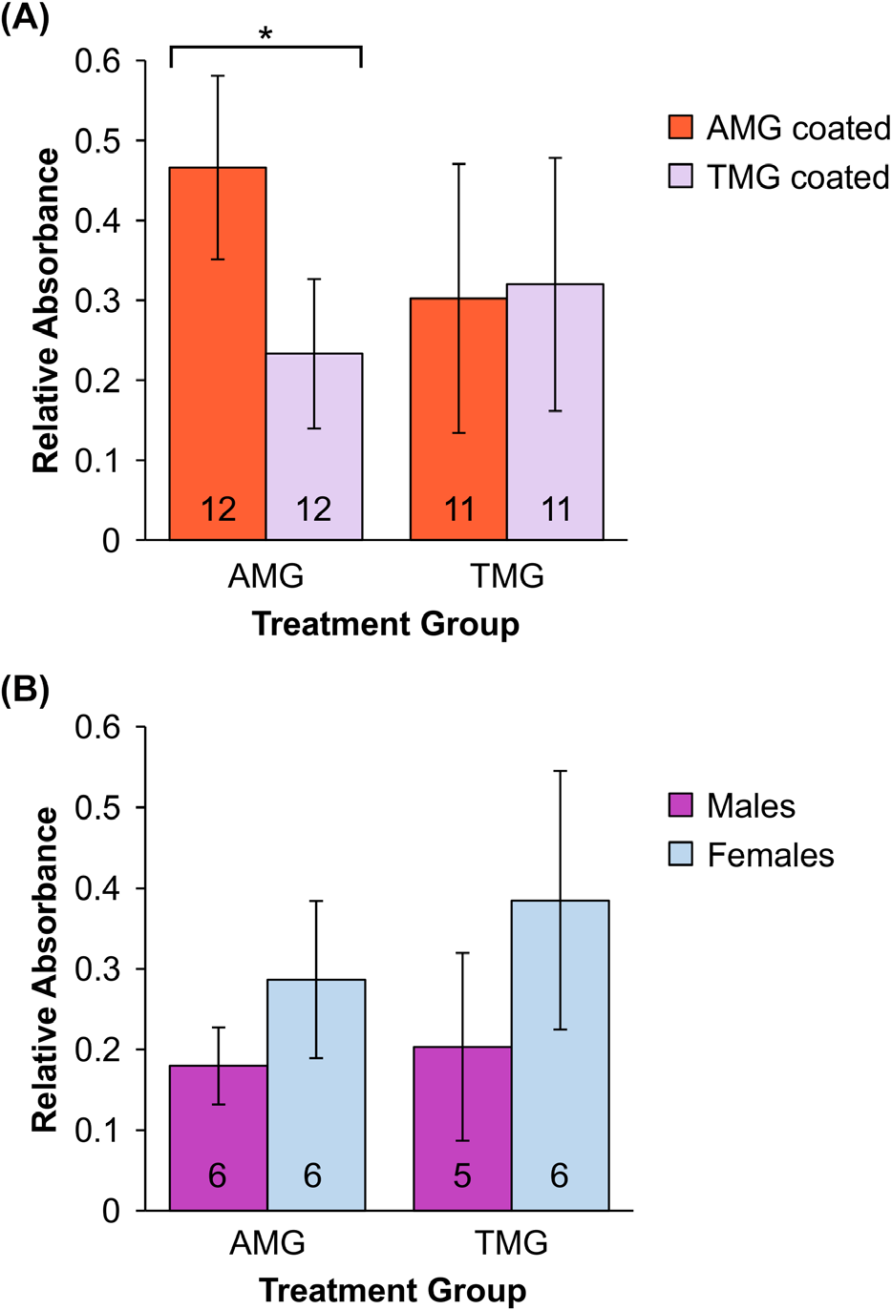
The immune response to the nanoparticle differs between treatments, but is similar for the PRRSV epitope. Vaccine candidates used for immunization of the mice are composed of the M‐GP5 epitope from porcine reproductive and respiratory syndrome virus (PRRSV) genetically fused to either the modified lumazine synthase from *Aquifex aeolicus* (AMG) or the modified tobacco mosaic virus coat protein (TMG). (A) Mean antibody responses to AMG and TMG vaccine candidates detected using enzyme‐linked immunosorbent assay (ELISA) plates coated with AMG (orange) or TMG (violet) antigen. AMG treatment shows a significantly higher response to the full antigen compared to the epitope alone (AMG and TMG coating, respectively; *P*‐value < 0.05). TMG treatment shows no clear difference in relative absorbance between plate coatings, indicating most antibodies are targeting the M‐GP5 epitope. (B) Mean epitope‐specific antibody responses by sex, with males and females shown in purple and blue, respectively. No significant difference was found between sexes for either treatment group (*P*‐values > 0.05). All data shown are from the 8000‐times dilution, error bars represent standard error, and significance was assessed using a Welch two sample *t*‐test. Numbers inside the bars represent sample sizes.

The immune response to TMG was specific to the M‐GP5 epitope as similar absorbance values were obtained whether the ELISA plates were coated with TMG or with AMG antigen (*P*‐value = 0.8109) (Fig. 8A). This indicates that the TMVc scaffold did not induce an immune response against itself. In contrast, the antibody response to AMG was not entirely specific to the M‐GP5 epitope. ELISA absorbances were significantly higher when the plates were coated with AMG than when they were coated with TMG (*P*‐value = 3.6 × 10^−5^), indicating that AaLSm is intrinsically immunogenic.

We included both male and female mice in this study because sex differences have been documented for the immune function of many animals, with females generally investing more towards adaptive and humoral responses compared to males [38, 39]. Historically, most animal trials either only include males or fail to investigate sex differences [40, 41]. This limits the scope and resulting interpretation of these studies because the test groups do not accurately represent the population, or sex‐dependent findings may be overlooked [40]. To avoid these biases and limitations, we investigated sex differences in the immune response to both vaccine candidates (Fig. 8B). Although there was no significant difference between the male and female mice for either AMG or TMG treatments (*P*‐values = 0.0615 and 0.0810, respectively), the females in both groups appear to have slightly higher antibody responses. Therefore, no clear sex difference was observed for these vaccine candidates.

## Discussion

Most recombinant protein biopharmaceuticals are currently produced in either bacteria or mammalian cells [29, 42]. An alternative to these platforms that has gained traction in recent years is the use of plants. While plants may not currently be the industry standard for recombinant protein production, this field is certainly on the rise with many new products in development, clinical trials, or use [28, 43]. This study demonstrates for the first time that AaLS and AaLSm have been produced in plants. We found that apoplast‐targeted AaLSm accumulates to relatively high soluble levels approaching 0.2 mg·g^−1^ of plant fresh weight, and that genetic fusion of the PRRSV M‐GP5 epitope does not significantly affect these levels. Previous work producing wild‐type AaLS in *E. coli* found high accumulation; however, it was almost completely insoluble [44]. In contrast, other studies have had more success producing soluble AaLS‐based nanoparticles in *E. coli* [19, 45]. In plants, the eukaryotic protein folding machinery and post‐translational modifications within the secretory pathway likely contribute to the relatively high soluble protein accumulation observed in this study [46, 47, 48]. We have also previously shown that plant production of a modified TMVc VLP showed high soluble protein accumulation with no significant change following genetic fusion of this PRRSV epitope [32].

We have demonstrated that plant‐produced AMG assembles into characteristic nanoparticle structures displaying the M‐GP5 epitope, and we had previously reported that TMG assembles into nanorod structures displaying the same M‐GP5 epitope [32]. Nanoparticle‐based vaccines have repetitive, multivalent epitope presentation that mimics the display of antigens on the surface of pathogens. This allows for enhanced antigen recognition and interaction with immune cells, resulting in stronger humoral responses compared to individual subunits alone [17, 49, 50]. The size of these nanoparticles contributes to the enhanced immune recognition as well. Particles 10–200 nm in diameter are efficiently filtered into lymphatic tissues containing high concentrations of antigen presenting cells, improving the stimulation of T and B cell responses [17].

During PRRSV infection, the immature virions pass through the secretory pathway, allowing the glycosylated envelope proteins to acquire complex N‐linked glycans characteristic of mammalian late‐Golgi processing [51]. Here we targeted recombinant AMG and TMG to the apoplast to facilitate complex *N*‐glycan processing and obtain a similar *N*‐glycan profile. Despite apoplastic purification, we report that both secreted AMG and TMG carry predominantly oligomannose *N*‐glycans on the GP5 ectodomain instead of complex *N*‐glycans with terminal GlcNAc residues and modifications with xylose as well as fucose. This glycosylation pattern is most often associated with proteins retained in the ER. Although these results were unexpected, there have been other reports of plant‐made viral glycoproteins retaining oligomannose glycans despite secretion to the apoplast. Margolin *et al*. [52] compared the glycosylation of proteins from the Marburg virus and human immunodeficiency virus (HIV) produced in *N. benthamiana* and human embryonic kidney cells 293 (HEK293). The plant‐produced proteins exhibited low rates of glycosylation, with minimal evidence of complex *N*‐glycans characteristic of Golgi processing and higher proportions of oligomannose *N*‐glycans. A study by Smargiasso *et al*. [53] also found that a secreted, soluble form of glycoprotein B from human cytomegalovirus produced in *Nicotiana tabacum* Bright Yellow‐2 (BY‐2) cell suspensions showed heterogeneous *N*‐glycans, with several specific *N*‐glycosylation sites predominantly displaying oligomannose *N*‐glycans. The presence of oligomannose *N*‐glycans on secreted AMG and TMG may be explained by (a) limited accessibility of the *N*‐glycans to further processing, (b) recombinant protein expression overloading the glycosylation machinery, or (c) as a result of unconventional secretion with bypassing of the Golgi apparatus [52, 53, 54, 55].

Upon subcutaneous injection into mice, both AMG and TMG induced antibody responses against the PRRSV epitope, with endpoint titers of 32 000 and 128 000‐times dilution, respectively. These results were only observed for the final bleeds, with lower antibody titers present in the test bleeds. This indicates that three doses were required for these vaccine candidates to induce robust immune responses. Xiong *et al*. [56] tested a PRRSV vaccine candidate composed of a truncated PRRSV GP5 genetically fused to *Salmonella* Typhimurium flagellin *fljB*. They found small increases in antibody production after two doses and greater increases following a third dose; however, their final titers were lower than those observed in the present study. Lu *et al*. [57] created four VLP vaccine candidates by fusing epitopes from PRRSV GP3 and GP5 to the hepatitis B core antigen. The specific antibody response in vaccinated mice gradually increased with time following multiple vaccine doses, showing similar final titers to those presented in the current study. Strategies to improve the immunogenicity of the AMG and TMG vaccine candidates may include altering the PRRSV epitope sequence, testing different adjuvants [58], or evaluating the effect of glycoengineering strategies to alter the epitope's glycan composition [59].

The purified protein samples administered to the mice predominantly contained the recombinant proteins; however, these samples were not entirely pure and retained minor amounts of endogenous plant proteins. For example, these samples appeared to contain the large subunit of RuBisCO, which is the most abundant protein in plant leaves and is notoriously difficult to completely remove during purification [60]. Because these samples were also used to coat the ELISA plates, it is possible that a small portion of the observed antibody responses were against these endogenous plant proteins instead of the vaccine candidates. However, this would only constitute a small fraction of the ELISA signal as the contaminating plant proteins were present in much lower quantities than the recombinant proteins, which were predominantly visible on stained gels.

Interestingly, most antibodies produced following TMG treatment target the PRRSV epitope, whereas AMG treatment resulted in antibodies against both the epitope and nanoparticle scaffold. Co‐administration of the tobacco mosaic virus (TMV) with a vaccine antigen has been shown to have adjuvanting effects, significantly increasing antibody production without inducing antibodies against itself [61]. In contrast, other plant viruses like the cauliflower mosaic virus and potato virus X result in virus‐specific antibody production as well [61]. For AaLS, a helper T‐cell epitope has been identified that can effectively bind immune cell receptors to enhance both humoral and cellular responses [62]. A similar protein nanoparticle composed of the lumazine synthase from *Brucella* spp. is reported to be immunogenic and have adjuvanting properties [63]. Further work is required to determine if AaLS nanoparticles show similar adjuvanting properties as the lumazine synthase from *Brucella* spp. and whether the helper T‐cell epitope is involved in this process.

Livestock vaccines have an important role in animal welfare, agriculture industries, mitigating climate impacts, and reducing pandemic risk for humans [64]. Veterinary pharmaceuticals follow many similar requirements as for humans; however, production costs are of particular importance for livestock vaccines because farmers are unlikely to adopt expensive products regardless of efficacy [25]. In contrast to human therapeutics which may cost dollars per dose, veterinary vaccines must cost a fraction of that to be economically viable [25, 65]. Furthermore, the majority of production costs for recombinant protein biopharmaceuticals arise from the downstream processing and purification steps [25]. Oral administration of lyophilized plant tissue containing the recombinant protein of interest would bypass these steps and greatly reduce production costs [66]. The storage, transport, and administration of these biopharmaceuticals would also benefit as proteins in lyophilized plant tissue are stable at room temperature for long durations [66, 67]. Oral administration also facilitates direct stimulation of the mucosal immune system. Injected vaccines induce systemic responses, mainly resulting in high blood IgG levels. In contrast, stimulating the mucosal immune system in the intestinal, reproductive, or respiratory tracts induces higher rates of IgAs across these tissues [67, 68]. PRRSV most commonly infects through the respiratory and reproductive tracts [69], therefore mucosal IgAs would be more effective at protecting animals [67, 70]. Testing and optimizing oral administration are beyond the scope of this study, however, they are future goals for this work.

## Materials and methods

### Construct design and cloning

The *AaLS* sequence encodes the entire AaLS monomer (accession: WP_010880027.1), and *AaLSm* encodes the same sequence with an N102Q amino acid substitution to remove the one potential N‐linked glycosylation site. For AMG, the PRRSV epitope includes residues 2–18 of M (accession: AAO13197.1) and 30–54 of GP5 (accession: AAO13196.1) from the PRRSV‐2 reference strain VR‐2332. The GP5 sequence also contains a two amino acid substitution (N33A and N51A) as described by Ansari *et al*. [35] The M and GP5 sequences are separated from each other and the C terminus of AaLSm by flexible (GGS)_4_ linkers. For the mouse immunization trial, TMG was similarly designed using a modified tobacco mosaic virus coat protein sequence as described by VanderBurgt *et al*. [32].

All genes were codon optimized for *N. benthamiana* nuclear expression and synthesized by Bio Basic Inc. (Markham, Canada) including flanking *Bsa*I sites for cloning. The genes were cloned into the pCLGGX‐plant protein expression vectors, which target the proteins to either the apoplast, vacuole, or ER [32], using Golden Gate cloning methodology [71]. The pCLGGX vectors all contain a PR1b signal peptide from tobacco to target the proteins for secretion [72]. The ER‐targeting vector also contains a KDEL tetrapeptide for ER retention [73], and the vacuole‐targeting vector contains a C‐terminal propeptide (CTPP) vacuolar sorting signal from the tobacco chitinase gene [74]. The regulatory elements within the pCLGGX‐vectors were described previously [32], and these plasmids were derived from pCAMterX [75]. Plasmid and gene sequences are available in Fig. S1. Plasmids were transformed into chemically competent *Agrobacterium tumefaciens* EHA105 cells.

### Recombinant protein expression

Wild‐type *N. benthamiana* plants were grown as described by VanderBurgt *et al*. [32], and infiltrations were performed as previously described [76]. Briefly, *Agrobacterium* cultures containing each plasmid of interest were co‐infiltrated with a culture containing *p19*, a suppressor of plant post‐transcriptional gene silencing from the *Cymbidium* ringspot virus [77]. The abaxial surface of three leaves of five 6–8‐week‐old *N. benthamiana* plants was infiltrated using a needleless syringe. *Agrobacterium* cultures containing *p19* were infiltrated alone as a negative control. For each protein, three leaf discs were collected per plant at 7 dpi and flash‐frozen in liquid nitrogen. This experiment was repeated three times for a total of 15 biological replicates.

For later experiments involving protein purification, vacuum infiltration was used [78]. Briefly, *N. benthamiana* plants were inverted and the leaves were submerged in a 1 : 1 : 1 mixture of water and *Agrobacterium* cultures (initial OD_600_ between 0.8 and 1.4) containing the gene of interest and *p19*. Negative pressure was applied to approximately −80 kPa, held for 1 min, and released slowly causing the *Agrobacterium* to infiltrate the leaves.

### SDS/PAGE and western blotting

Protein extraction from leaf tissue, SDS/PAGE, and western blotting were performed as previously described [32, 76]. Staining of gels or blotting membranes was conducted using GelCode Blue (Thermo Fisher, Burlington, ON, Canada, Cat. No. 24590) per the manufacturer's instructions.

### Enzymatic deglycosylation

Crude plant extracts were deglycosylated by incubating samples at 37 °C for 60 min with PNGase F (New England BioLabs, Whitby, ON, Canada, Cat. No. P0704L) as per the manufacturer's instructions. Negative controls consisted of the same treatment lacking PNGase F. All samples were analyzed by SDS/PAGE and western blotting.

### Protein purification

A primer‐based approach was used to add a sequence encoding a 6x His‐tag to the 3′ end of both the *AaLSm* and *AMG* genes following the c‐Myc tag sequence. Primers were designed to amplify the genes from the expression plasmids, add the His‐tag sequence, and introduce Bsa*I* sites for cloning. After PCR amplification, the samples were cloned back into the apoplast‐targeting pCLGG‐X vector and sequenced to confirm no mutations were introduced. Plasmids were transformed into chemically competent *Agrobacterium*.

Frozen infiltrated plant tissue was homogenized using a cold mortar and pestle, with 10× v/w of extraction buffer [76] containing 5 mm imidazole. The homogenate was sonicated using a Q500 Sonicator (Qsonica, Newtown, CT, USA) at 40% amplitude for 5 min in 30 s pulses, followed by centrifugation at 20 000 ***g*** for 10 min at 4 °C. The resulting supernatant was filtered through Miracloth (Millipore Sigma, Oakville, ON, Canada, Cat. No. 475855). AMG samples were clarified by heat treatment in a 60 °C water bath for 10 min, whereas TMG samples were clarified by incubation at 4 °C overnight. Following a second centrifugation step at 20 000 ***g*** at either room temperature or 4 °C, the extracts were filtered again.

IMAC was performed using Ni Sepharose 6 Fast Flow resin (Cytiva, sold by Millipore Sigma, Oakville, ON, Canada, Cat. No. 17531801) as per the manufacturer's instructions for batch and gravity‐flow purification. Briefly, 1 mL of resin was added to a 10 mL column (Bio‐Rad, Mississauga, ON, Canada, Cat. No. 7311550), rinsed with water, and equilibrated with 1× PBS containing 5 mm imidazole. The resin was transferred into the plant extract and incubated for 1 h at either room temperature or 4 °C, with gentle agitation. The extracts were centrifuged at 1000 ***g*** for 2 min to pellet the resin, and most of the supernatant was removed by aspiration. The resin was resuspended in the remaining extract, moved back into the column, and the remaining liquid was allowed to drip through. The resin was washed twice with 5 mL wash buffer (1× PBS, 20 mm imidazole). Bound proteins were eluted from the resin using 5 mL of 1× PBS containing 500 mm imidazole, collected in 1 mL fractions.

Size‐exclusion chromatography (SEC) was performed with a Superose 6 Increase 10/300 GL column (GE Healthcare, Mississauga, ON, Canada) using an NGC Quest 10 Plus Chromatography System (Bio‐Rad). A running buffer of 1× PBS was used, 0.5 mL of IMAC‐purified samples were loaded, and 0.5 mL fractions were collected.

### Apoplastic fluid isolation

Apoplastic fluid isolation was performed as previously described [79], except a buffer composed of 20 mm 2‐(*N*‐morpholino)ethanesulfonic acid (MES), 2 mm CaCl_2_, 0.1 m NaCl, pH 6 was used. Briefly, infiltrated *N. benthamiana* leaves were sampled at 7 dpi. The leaves were submerged in the apoplast isolation buffer and vacuum infiltrated twice. After drying the external surfaces of the leaves, they were rolled up, placed into a 30 mL syringe inside a 50 mL tube, and centrifuged at 2100 ***g*** for 3 min. The resulting solution was centrifuged again at 16 000 ***g*** for 5 min at 4 °C to pellet any insoluble material. The supernatant was collected and stored on ice until use.

### Liquid chromatography‐mass spectrometry

IMAC‐purified AMG and TMG from either whole cell lysate or apoplastic fluid isolation were resolved by SDS/PAGE, and bands corresponding to the recombinant protein monomers were excised from the gel as described previously [80]. The method for protein digestion and performing LC–MS has also been previously described [81]. Proteins were digested with trypsin (Promega, Walldorf, Germany) and chymotrypsin (Promega) and analyzed by LC–MS. The digested samples were loaded on a nanoEase C18 column (nanoEase M/Z HSS T3 Column, 100 Å, 1.8 μm, 300 μm × 150 mm, Waters) using 0.1% formic acid as the aqueous solvent. A gradient from 1% B (B: 80% Acetonitrile, 0.1% FA) to 40% B in 50 min was applied, followed by a 10 min gradient from 40% B to 95% B that facilitates elution of large peptides, at a flow rate of 6 μL·min^−1^. Detection was performed with an Orbitrap MS (Exploris 480, Thermo) equipped with the standard H‐ESI source in positive ion, DDA mode (= switching to MSMS mode for eluting peaks). MS scans were recorded and the highest peaks were selected for fragmentation. The possible glycopeptides were identified as sets of peaks consisting of the peptide moiety and the attached *N*‐glycan varying in the number of HexNAc units, hexose, deoxyhexose, and pentose residues. The theoretical masses of these glycopeptides were determined with a spreadsheet using the monoisotopic masses for amino acids and monosaccharides. Manual glycopeptide searches were made using freestyle 1.8 (Thermo).

### Transmission electron microscopy and immunogold localization

Transmission electron microscopy (TEM) was used to visualize nanoparticle assembly using SEC‐purified samples. TEM and immunogold localization were performed as previously described [32]. All grids were examined on a JEM‐1400 TEM (JEOL Canada Inc, Saint‐Hubert, QC, Canada) operated at 80 kV.

### Ethics statement

The animal research in this study was reviewed and approved by the Animal Care Committee at the University of Western Ontario, London, ON, Canada (Protocol 2023‐032). The study design, animal handling, and experimental treatments followed policies and guidelines from the Canadian Council on Animal Care.

### Mouse immunization trial

The first two IMAC elutions were combined and concentrated using Vivaspin 500 10 kDa MWCO columns (Cytiva, Cat. No. 28932225), as per manufacturer's instructions. The samples then underwent sequential buffer‐exchange steps in 1× PBS containing decreasing concentrations of imidazole (250, 125, 65, and 0 mm) and were stored at −20 °C until use. Sample preparation following IMAC was identical for both proteins used in the immunization trial.

The immunogenicity of the two vaccine candidates was evaluated using 5‐week‐old BALB/c mice (Charles River) housed within the University of Western Ontario's Animal Care and Veterinary Services buildings. The animal research in this study was reviewed and approved by the Animal Care Committee at the University of Western Ontario, London, ON, Canada (Protocol 2023‐032). The study design, animal handling, and experimental treatments followed policies and guidelines from the Canadian Council on Animal Care. The mice also had access to food and water *ad libitum* throughout the study.

The mice were separated into three treatment groups and were administered either AMG, TMG, or PBS as a negative control. All treatment groups consisted of 6 male and 6 female mice, except for TMG males and PBS females that each had 5 mice. For every dose, mice were subcutaneously injected with 100 μL of a 1 : 1 mixture of adjuvant with either PBS or 33 μg of antigen in PBS. Freund's complete adjuvant was used in the primary immunization, and Freund's incomplete adjuvant was used for both booster doses.

Pre‐immune blood samples were taken by lateral tail vein on day 0 before the primary injection on day 1. The first booster dose was administered on day 13 followed by a test bleed on day 27. A second booster dose occurred on day 34, and the final bleed was taken intracardially from anesthetized mice on day 47 (Fig. 7B). Mice were sacrificed at this experimental endpoint. Following each sampling event, blood samples were kept at 4 °C overnight then centrifuged at 1000 ***g*** for 10 min. The serum was separated from precipitated blood components, aliquoted, and stored at −80 °C.

### ELISAs for antibody response

IMAC‐purified AMG or TMG were used to coat 96‐well plates (Thermo Fisher, Cat. No. 44‐2404‐21) overnight at 4 °C at a concentration of 2 μg·mL^−1^ in a 0.1 m carbonate–bicarbonate buffer, pH 9.4. Detection using plates coated with the administered antigen facilitated the detection of antibodies recognizing any component of the vaccine candidate, whereas coating with the opposite protein allowed the detection of epitope‐specific antibodies. The plates were washed three times with 1× PBS, 0.05% Tween 20 before blocking in 2% bovine serum albumin (BSA) in PBS for 2 h or overnight. The plates were then washed three times.

Serial dilutions of mouse sera were performed using 1% BSA‐PBS, and a commercial mouse anti‐c‐Myc antibody (GenScript, Piscataway, NJ, USA Cat. No. A00864) was similarly diluted as both a standard and positive control. Two technical replicates were included for each sample, which were added to the plates and incubated overnight at 4 °C. The plates were washed thrice, then incubated for 1 h in a 1/5000 dilution of goat anti‐mouse horseradish peroxidase‐conjugated secondary antibody (Bio‐Rad, Cat. No. 1706516) in 1% BSA‐PBS. The plates were washed again three times followed by a 15–20 min incubation in 2,2′‐azino‐bis(3‐ethylbenzothiazoline‐6‐sulfonic acid) (ABTS) containing 1 μL·mL^−1^ of 30% hydrogen peroxide. Plates were analyzed using an iMark Microplate Absorbance Reader (Bio‐Rad) at 415 nm wavelength.

### Data analyses

Analysis of the recombinant protein accumulation was performed as previously described by VanderBurgt *et al*. [32]. Accumulation levels for all 15 biological replicates were examined using r 4.2.3 [82], and significance was assessed using Welch two sample *t*‐test.

For the ELISA data, a standard curve was created for each plate using the anti‐c‐Myc antibody samples as a standard curve to calculate the relative absorbance values against. The method described by Frey *et al*. [83] was used to determine the endpoint titer for the final bleeds of both vaccine groups. Using this method, a threshold value was calculated for each dilution using the mean relative absorbance from the PBS control final bleeds, plus 1.893 times the respective standard deviation. The multiplication factor of 1.893 is based on a confidence level of 95% with 11 control samples [83]. The endpoint titers were determined as the greatest dilution where the treatment group's mean relative absorbance remained above that dilution's threshold value.

Additional statistical analyses were performed for the ELISA data using r 4.2.3 [82] to compare the antibody response by sex, as well as the response against the epitope or full antigen. Significance was assessed using Welch two sample *t*‐test.

## Conflict of interest

The authors declare no conflict of interest.

## Author contributions

JTVB designed the constructs, conducted most of the experiments and analyses, and wrote the manuscript. RS performed the mass spectrometry experiment and analysis. CPG conceptualized the epitope and the AaLS nanoparticle. AK assisted with protein purification, and HZ assisted with ELISAs. RM conceptualized the study and edited the manuscript. All authors contributed to the article and approved the submitted version.

## Supporting information


**Fig. S1.** Expression construct and gene of interest nucleotide and mature protein sequences.

## Data Availability

Supporting Information is available containing nucleotide and mature protein sequences for the expression plasmids and genes of interest. The data that support the findings of this study are available in the figures and/or the Supporting Information of this article.
