# Supplementary material for: Protein nanoparticles assemble in plants, display antigenic viral peptides, and produce an epitope‐specific immune response
Source: FEBS J. 2025 Nov 17;293(7):1891–907. doi: 10.1111/febs.70288 (PMC13044993; doi:10.1111/febs.70288)
Supplement: Supplementary file 1 — Fig. S1. Expression construct and gene of interest nucleotide and mature protein sequences. [file FEBS-293-1891-s001.pdf]

Supplementary Information

Figure S1. Expression construct and gene of interest nucleotide and mature protein sequences. For the **(A)** nucleotide and **(B)** mature protein sequences, an individual legend of colour-representations are shown. The individual legend for the nucleotide sequences in **(A)** also begins with a schematic of the regulatory elements within the plasmid.

(A) Nucleotide Sequences

Legend:

2x35S	tCUP	PR1b	GOI and tag coding region	NosT

HA tag c-Myc tag 6x His tag* AaLS AaLSm TMVc M sequence
GP5 sequence linker **start codon** nt from plasmid cloning **stop codons**

**Note:* the 6x His tag is in brackets for AaLSm and AMG to represent the protein with and without this tag's coding sequence introduced to the 3' end of each gene.

AaLSm-M-GP5 (AMG) in expression plasmid (apoplast-targeting)

AAGCTTGCCAACATGGTGGAGCACGACACTCTCGTCTACTCCAAGAATATCAAAGATACAGTCTCAGAAG
ACCAAAGGGCTATTGAGACTTTTCAACAAAGGGTAATATCGGGAAACCTCCTCGGATTCCATTGCCCAGC
TATCTGTCACTTCATCAAAAGGACAGTAGAAAAGGAAGGTGGCACCTACAAATGCCATCATTGCGATAAA
GGAAAGGCTATCGTTCAAGATGCCTCTGCCGACAGTGGTCCCAAAGATGGACCCCCACCCACGAGGAGCA
TCGTGGAAAAAGAAGACGTTCCAACCACGTCTTCAAAGCAAGTGGATTGATGTGAACATGGTGGAGCACG
ACACTCTCGTCTACTCCAAGAATATCAAAGATACAGTCTCAGAAGACCAAAGGGCTATTGAGACTTTTCA
ACAAAGGGTAATATCGGGAAACCTCCTCGGATTCCATTGCCCAGCTATCTGTCACTTCATCAAAAGGACA
GTAGAAAAGGAAGGTGGCACCTACAAATGCCATCATTGCGATAAAGGAAAGGCTATCGTTCAAGATGCCT
CTGCCGACAGTGGTCCCAAAGATGGACCCCCACCCACGAGGAGCATCGTGGAAAAAGAAGACGTTCCAAC
CACGTCTTCAAAGCAAGTGGATTGATGTGATATCTCCACTGACGTAAGGGATGACGCACAATCCCACTAT
CCTTCGCAAGACCCTTCCTCTATATAAGGAAGTTCATTTCATTTGGAGAGGACACGCTGAAATCACCAGT
CTCTCTCTACAAATCTATCTCTCTCGAGCTTTCGCGAGCTCGACTCTAGGGGATCCGATCCGATCTATCC
TCTTATCTCTCAAACTCTCTCGAACCTTCCCCTAACCCTAGCAGCCTCTCATCATCCTCACCTCAAAACC
CA**ATG**GGATTTTTCTCTTTTCACAAATGCCCTCATTTTTCTTGTGTCGACACTTCTCTTATTCCTAAT
AATATCTCACTCTTCTCATGCCGGCGGTTACCCATACGATGTGCCTGATTACGCTCAGATCTATGAAGGT
AAATTGACTGCTGAAGGTCTTAGATTTGGTATTGTTGCTTCTCGGTTTAATCATGCTCTTGTTGATAGAC
TTGTGGAAGGTGCTATTGATTGTATTGTTAGGCACGGCGGTCGTGAGGAAGATATAACACTAGTTCGGGT
CCCTGGTTCTTGGGAGATTCCTGTTGCTGCTGGTGAACTTGCTAGAAAGGAAGATATTGATGCTGTTATA
GCTATTGGTGTTCTTATTAGAGGTGCTACTCCTCATTTTGATTATATTGCTAGTGAAGTTTCTAAGGGTC
TTGCTCAACTTTCTCTTGAACTTAGAAAGCCTATTACTTTTGGTGTTATTACTGCTGATACTCTTGAACA
AGCTATTGAAAGAGCTGGTACTAAGCATGGTAATAAGGGTTGGGAAGCTGCTTTGTCTGCTATTGAAATG
GCTAATCTTTTTAAGTCTCTTAGAGGTGGTTCAGGTGGTTCTGGTGGTAGTGGAGGTAGTGGTTCCTCTC
TTGATGATTTTTGCCATGATTCTACCGCTCCTCAAAAGGTTGGTGGTTCAGGTGGTTCTGGTGGTAGTGG
AGGTAGTAATGCTAGTGCTGACTCTAGTTCACACCTCCAGCTCATCTATAATTTGACTCTTTGTGAACTT
GCTGGTACTGATGAGCAAAAGTTGATCTCTGAGGAAGATCTT(CACCATCATCATCATCAT)**TGATAG**AA
TTCGGTACCGAGCTCGAATTTCCCCGATCGTTCAAACATTTGGCAATAAAGTTTCTTAAGATTGAATCCT
GTTGCCGGTCTTGCGATGATTATCATATAATTTCTGTTGAATTACGTTAAGCATGTAATAATTAACATGT
AATGCATGACGTTATTTATGAGATGGGTTTTTATGATTAGAGTCCCGCAATTATACATTTAATACGCGAT
AGAAAACAAAATATAGCGCGCAAACTAGGATAAATTATCGCGCGGTGTCATCTATGTTACTAGATC

The following sequences are present in place of AMG in the above schematic:

AaLS sequence

GGCGGTTACCCATACGATGTGCCTGATTACGCTCAGATCTATGAAGGTAAATTGACTGCTGAAGGTCTTA
GATTTGGTATTGTTGCTTCTCGGTTTAATCATGCTCTTGTTGATAGACTTGTGGAAGGTGCTATTGATTG
TATTGTTAGGCACGGCGGTCGTGAGGAAGATATAACACTAGTTCGGGTCCCTGGTTCTTGGGAGATTCCT
GTTGCTGCTGGTGAACTTGCTAGAAAGGAAGATATTGATGCTGTTATAGCTATTGGTGTTCTTATTAGAG
GTGCTACTCCTCATTTTGATTATATTGCTAGTGAAGTTTCTAAGGGTCTTGCTAATCTTTCTCTTGAACT
TAGAAAGCCTATTACTTTTGGTGTTATTACTGCTGATACTCTTGAACAAGCTATTGAAAGAGCTGGTACT
AAGCATGGTAATAAGGGTTGGGAAGCTGCTTTGTCTGCTATTGAAATGGCTAATCTTTTTAAGTCTCTTA
GAGAGCAAAAGTTGATCTCTGAGGAAGATCTT**TGATAG**

AaLSm sequence

GGCGGTTACCCATACGATGTGCCTGATTACGCTCAGATCTATGAAGGTAAATTGACTGCTGAAGGTCTTA
GATTTGGTATTGTTGCTTCTCGGTTTAATCATGCTCTTGTTGATAGACTTGTGGAAGGTGCTATTGATTG
TATTGTTAGGCACGGCGGTCGTGAGGAAGATATAACACTAGTTCGGGTCCCTGGTTCTTGGGAGATTCCT
GTTGCTGCTGGTGAACTTGCTAGAAAGGAAGATATTGATGCTGTTATAGCTATTGGTGTTCTTATTAGAG
GTGCTACTCCTCATTTTGATTATATTGCTAGTGAAGTTTCTAAGGGTCTTGCTCAACTTTCTCTTGAACT
TAGAAAGCCTATTACTTTTGGTGTTATTACTGCTGATACTCTTGAACAAGCTATTGAAAGAGCTGGTACT
AAGCATGGTAATAAGGGTTGGGAAGCTGCTTTGTCTGCTATTGAAATGGCTAATCTTTTTAAGTCTCTTA
GAGAGCAAAAGTTGATCTCTGAGGAAGATCTT(CACCATCATCATCATCAT)**TGATAG**

TMVc-M-GP5 (TMG) sequence

GGCGGTTACCCATACGATGTGCCTGATTACGCTTCTTACTCTATTACTACTCCTTCTCAATTTGTTTTTC
TTTCTTCTGCTTGGGCTGATCCTATTGAATTGATTAATCTTTGTACTAATGCTCTTGGTAATCAGTTTCA
AACTCAACAAGCTAGGACTGTTGTTCAAAGACAATTTTCTCAAGTTTGGAAGCCTTCTCCACAAGTTACT
GTTAGATTTCCTGATTCTGATTTTAAGGTTTACAGATATAATGCTGTTTTGAATCCTCTTGTTACTGCTT
TGCTTGGAGCTTTTGATACTAGAAATAGGATTATTGAAGTTGAAAATCAAGCTAATCCAACTACTGCTGA
AACTCTTGATGCTACAAGAAGAGTTGATGATGCTACTGTTGCTATTAGGTCTGCTATTAATAACCTTATT
GTTGAACTTATTAGGGGTACTGGTTCTTATAATAGGTCATCTTTTGAATCTTCTTCTGGTCTTGTTTGGA
CTTCTGGTCCTGCTACTGGTGGTTCAGGTGGTTCTGGTGGTAGTGGAGGTAGTGGTTCCTCTCTTGATGA
TTTTTGCCATGATTCTACCGCTCCTCAAAAGGTTGGTGGTTCAGGTGGTTCTGGTGGTAGTGGAGGTAGT
AATGCTAGTGCTGACTCTAGTTCACACCTCCAGCTCATCTATAATTTGACTCTTTGTGAACTTGCTGGTA
CTGATGAGCAAAAGTTGATCTCTGAGGAAGATCTTCACCATCATCATCATCAT**TGATAG**

(B) Mature Protein Sequences

Legend:

HA tag c-Myc tag 6x His tag* AaLS AaLSm TMVc

M sequence GP5 sequence linker "GG" added during cloning

**Note:* the 6x His tag is in brackets for AaLSm and AMG to represent the protein with and without this tag's coding sequence introduced to the 3' end of each gene.

AaLS

GGYPYDVPDYAQIYEGKLTAEGLRFGIVASRFNHALVDRLVEGAIDCIVRHGGREEDITLVRVPGSWEIP
VAAGELARKEDIDAVIAIGVLIRGATPHFDYIASEVSKGLANLSLELRKPITFGVITADTLEQAIERAGT
KHGNKGWEAALSAIEMANLFKSLREQKLISEEDL

AaLSm

GGYPYDVPDYAQIYEGKLTAEGLRFGIVASRFNHALVDRLVEGAIDCIVRHGGREEDITLVRVPGSWEIP
VAAGELARKEDIDAVIAIGVLIRGATPHFDYIASEVSKGLAQLSLELRKPITFGVITADTLEQAIERAGT
KHGNKGWEAALSAIEMANLFKSLREQKLISEEDL(HHHHHH)

AaLSm-M-GP5 (AMG)

GGYPYDVPDYAQIYEGKLTAEGLRFGIVASRFNHALVDRLVEGAIDCIVRHGGREEDITLVRVPGSWEIP
VAAGELARKEDIDAVIAIGVLIRGATPHFDYIASEVSKGLAQLSLELRKPITFGVITADTLEQAIERAGT
KHGNKGWEAALSAIEMANLFKSLRGGSGGSGGSGGSGSSLDDFCHDSTAPQKVGGSGGSGGSGGSNASAD
SSSHLQLIYNLTLCELAGTDEQKLISEEDL(HHHHHH)

TMVc-M-GP5 (TMG)

GGYPYDVPDYASYSITTPSQFVFLSSAWADPIELINLCTNALGNQFQTQQARTVVQRQFSQVWKPSPQVT
VRFPDSDFKVYRYNAVLNPLVTALLGAFDTRNRIIEVENQANPTTAETLDATRRVDDATVAIRSAINNLI
VELIRGTGSYNRSSFESSSGLVWTSGPATGGSGGSGGSGGSGSSLDDFCHDSTAPQKVGGSGGSGGSGGS
NASADSSSHLQLIYNLTLCELAGTDEQKLISEEDLHHHHHH
